# Supplementary material for: Proteins and DNA Sequences Interacting with Tanshinones and Tanshinone Derivatives
Source: Int J Mol Sci. 2025 Jan 20;26(2):848. doi: 10.3390/ijms26020848 (PMC11765770; doi:10.3390/ijms26020848)
Supplement: Supplementary file 1 [file ijms-26-00848-s001.zip › ijms-3412819-supplementary.pdf]

Supplementary Table 1 and 2

Table S1. Values of C_{max} in selected tanshinone pharmacokinetic studies after adinistration of tanshinones, tanshinone derivatives or *S. miltiorrhiza* (*S.m.*) extracts. Following abbreviations were used; Dihydrotanshinone I (DHTI), Cryptotanshinone (CT), Tanshinone I (TI), Tanshinone IIA (TIIA), Miltirone (MT), and Sodium Tanshinone IIA Sulphonate (STS).

**Animal studies**				
**Nr**	**Tested organism**	**Tanshinone dose**	**Pharmacokinetic parameters (C_{max}; (μg/L)**	**Reference**
1	Rats	Intragastric administration of *S.m.* ethanol extract (M-E, 0.65 g/kg), or combination of *S.m.* ethanol extract (M-WE, 0.65 g/kg) and *S.m.* water extract 1.55 g/kg.	**TIIA:**<br>M-E 25.46 ± 8.44 ,<br>M-WE 31.10 ± 3.64<br><br>**CT:**<br>M-E 47.64 ± 14.34,<br>M-WE 94.02 ± 12.89<br><br>**DHTI:**<br>M-E 50.29 ± 24.78,<br>M-WE 49.17 ± 24.37<br><br>**TI:**<br>M-E 11.22 ± 0.97,<br>M-WE 16.55 ± 0.94	20
2	Sprague-Dawley rats	Sprague-Dawley rats, orally administered with the CT dispersion (20 mg/kg). CT is metabolized in rats to TIIA.	**CT** 25.1 ± 6.5<br>**TIIA** approx. value 11.8	49
3	Male Sprague-Dawley rats	CT (16 mg/kg) orally administered in three forms: as suspension, solid lipid nanoparticles based on glyceryl monostearate (GMS-SLNs) and Compritol 888 ATO as CP-SLNs.	**CT:**<br>approximately<br>49.82-67.32, dependent on GMS-SLNs concentration 0-1%<br><br>53.68-73.45<br>dependent on CP-SLNs concentration 0-1%<br><br>suspension 20.89±1.96<br><br>**TIIA:**<br>7.17-7.78, dependent on GMS-SLNs concentration 0-1%<br><br>6.86-7.94<br>dependent on CP-SLNs concentration 0-1%<br><br>suspension 18.43±1.36	27
4	Male Sprague-Dawley rats	Pure TIIA, solid dispersions (SDs) and physical mixtures (PMs) of TIIA with silica nanoparticles administered orally (60 mg/kg).	**Pure TIIA** 72.78 ± 6.59<br>**SDs** 152.34 ± 16.28<br>**PMs** 76.1 ± 8.17	25
5	Male Sprague-Dawley rats	A single oral dose of the mixture: Danshensu 10.25 mg/kg, Rosmarinic acid 6.39 mg/kg, CT 9.82 mg/kg, DHTI 3.58 mg/kg, TI 3.90 mg/kg and TIIA 5.79 mg/kg.	**CT** 42.85 ± 13.58<br>**DHTI** 11.29 ± 11.49<br>**TI** 54.64 ± 17.72<br>**TIIA** 22.24 ± 3.42	50
6	Male Sprague-Dawley rats	TIIA and solid dispersion of TIIA and low molecular weight chitosan (LMC) at a weight ratio 1:3 – 1:12. Dose equivalent to 60 mg/kg TIIA, administered orally.	**TIIA:**<br>without LMC 64.06 – 5.492<br>with LMC 75.26 – 7.382	51

7	Male Sprague-Dawley rats	Mixture of herbs Huo Luo Xiao Ling Dan (HLXLD) and *S. miltiorhiza* extracts, both administered orally at doses equivalent to 1.26 g/kg.	HLXLD group:<br>**CT** 45.67 ± 4.26<br>**DHTI** 21.99 ± 3.70<br>**TI** 127.01 ± 43.10<br>**TIIA** 77.01 ± 22.43<br>**TIIB** 21.98 ± 2.41<br><br>*S. miltiorrhiza* group:<br>**CT** 36.09 ± 7.73<br>**DHTI** 15.29 ± 3.18<br>**TI** 100.86 ± 7.59<br>**TIIA** 72.99 ± 21.66<br>**TIIB** 16.58 ± 2.96	52
8	Male Sprague-Dawley rats	A standardized extract from *S. miltiorrhiza* roots PF2401-SF was orally administered at a dose of 10 mg/kg.<br>It is equivalent<br>to 1.15 mg/kg of TI, 1.10 mg/kg of DHTII, 4.1 mg/kg of TIIA and 1.91 mg/kg of CT.	**CT** 0.66 ± 0.27<br>**DHTI** 3.23 ± 1.40<br>**TI** 1.63 ± 0.78<br>**TIIA** 2.78 ± 0.96	53
9	Male Sprague-Dawley rats	Bushen Huoxue Qubi (BHQ) granules preparation composed of 10 medicinal herbs. The dose of the BHQ solution (60 mg/kg of TIIA) was administered by gastric gavage to animals.	**TIIA** 70.78±9.62	54
10	Male Sprague-Dawley rats	Oral administration of pure tanshinone monomers and the liposoluble extract of *S. miltiorrhiza*.<br><br>Group I, CT (n = 5, intragastric administration (i.g.), 34 mg/kg);<br>Group II, TIIA (n = 5, i.g., 70 mg/kg);<br>Group III, DHTI (n = 5, i.g., 18 mg/kg);<br>Group IV, TI (n = 5, i.g., 13 mg/kg);<br>Group V, *S. miltiorrhiza* extract (n = 5, i.g., 800 mg/kg, equivalent to 34 mg/kg CT, 70 mg/kg TIIA, 18 mg/kg DHTI and 13 mg/kg for TI with coexisting other tanshinones).	Pure tanshinones:<br>**CT** 48.8 ± 24.5<br>**DHTI** 20.0 ± 1.6<br>**TI** 3.4 ± 2.0<br>**TIIA** 10.1 ± 6.0<br><br>*S. miltiorrhiza* extract:<br>**CT** 235.6 ± 59.6<br>**DHTI** 34.6 ± 7.9<br>**TI** 8.5 ± 2.2<br>**TIIA** 48.9 ± 12.9	55
11	Male Sprague-Dawley rats	Oral administration of solid dispersions or physical mixtures TIIA with porous silica, at a dose of 60 mg/kg TIIA.	**TIIA:**<br>Physical mixtures 66.91 ± 16.982<br>Solid dipspersions 165.36 ± 28.503	56
12	Male Sprague-Dawley rats	Oral administartyion of MT (suspended in Tween 80) at a single oral dose of 20, 40 and 60 mg/kg. Twenty animals were used in the test.	**MT:**<br>40.79 ± 5.67 (dose 20 mg/kg)<br>92.70 ± 13.43 (dose 40 mg/kg)<br>144.90 ± 7.56 (dose 60 mg/kg)	57
13	Male Sprague-Dawley rats	CT (2 mg/kg) was administered orally to:<br>control group:<br>CT suspension with 20% PEG 300<br><br>solid dispersion 10 (SD10) group (CT:poloxamer 407 = 1:9, w/w)<br><br>solid dispersion 50 (SD50) group (CT:poloxamer 407 = 1:49, w/w)	**CT:**<br>SD10 group 3.9 ± 3.1<br>SD50 group 12.6 ± 5.80	26
**Human studies**				
**Nr**	**Tested organism**	**Tanshinone dose**	**Pharmacokinetic parameters (C_{max}, µg/L)**	**Reference**

1	Humans	A 40 mg dose of STS administered as intravenous infusion (0.16 mg/mL) over1 h	743.6 ± 0.1	58
2	Humans	A single oral dose of traditional decocotion (TD) or micronized preparation, both contained 20 g crude *S. miltiorrhiza*	TD:<br>**CT** 6.37 (1.26–26.70)<br>**TI** 0.43 (0.00–4.80)<br>**TIIA** 0.82 (0.32–2.52)<br><br>Micronized preparation:<br>**CT** 146.70 (14.40–301)<br>**TI** 6.57 (1.84–32.50)<br>**TIIA** 25.80 (3.76–48.30)	21

Table S2. Signaling pathways affected by tanshinones or tanshinone analogues in the *in vivo* tests on animals or humans. Following abbreviations were used; Tanshinone IIA (TIIA), and sodium tanshinone IIA sulphonate (STS).

Nr	Tanshinone	Animal treatment	Affected signaling route	Clinical outcome	References
1	TIIA administered *per os* 1.5 mg/kg	Rat model of heart failure, Sprague-Dawley male rats.	TLR4/ NF-κB p65	Inhibition of cardiomyocyte pyroptosis decreased histological cardiac tissue damage, lowered serum levels of NT-pro-BNP, IL-1β, and IL-18	150
2	TIIA administered *per os* 1.5 mg/kg	Rat model of heart failure. Sprague-Dawley male rats.	AMPK-Mtor	Inhibition of apoptosis and inducing autophagy via activation of the AMPK-Mtor	151
3	STS, intraperitoneal injection 10 mg/kg, i.p.)	Induction of endotoxemia via intraperitoneal (i.p.) injection 10 mg/kg of LPS to mice. Male mice-type C57BL/6.	NLRP3	Increased the survival rates, improved the cardiac function and reduced myocardial inflammatory injury	152
4	STS, intraperitoneal injections of 10 or 20 mg/kg	Rat model of injury-induced intervertebral disc degeneration. Male Sprague-Dawley rats.	P38 MAPK	Ameliorated injury-induced intervertebral disc degeneration	32
5	1 mg/kg or 10 mg/kg TIIA through intraperitoneal injection once a week	Spontaneously hypertensive male SHRs and Wistar-Kyoto (WKY) rats	Cys-C/Wnt	Decreased following parameters: SBP, HW/BW, LVW/BW, swelling degree of myocardial cells, COL1A1 and COL3A1 mrna expressions, serum cTnI and ADMA levels	153
6	STS (5 mg/kg, twice per day) by airway inhalation	Male C57BL/6J mice	MAPK/HIF-1a	Improved pulmonary function, ameliorated emphysema and decreased the infiltration of inflammatory cells in the lungs of cigarette smoke male mice	154
7	TIIA (50 mg/kg/day) in corn oil	C57BL/6 male mice	GSK3β and downstream - activated protein kinases	Ameliorated ischemia/reperfusion-induced damage and protection against the transition of acute kidney injury to chronic kidney disease	155

8	STS, 10 mg/kg/day, injected intraperitoneally	Wild-type C57BL/6J male mice	ERK1/2 and NF-κB	Amelioration of pulmonary inflammatory response, and lung function decline in mice exposed to cigarette smoke and LPS	156
9	STS 30 mg/kg/d, injected intraperitoneally	Adult male Sprague-Daley rats	BMP9-BMPR2-Smad1/5/9	Significant inhibition of the hypoxia-induced apoptosis in the pulmonary endothelium	157
10	STS, intraperitoneally injected at a dose of 5 or 10 mg/kg and repeated again after 4 hrs	Male C57BL/6 mice	NF-κB and IFN-γ/STAT1	STS protects mice against concanavalin A-induced hepatitis	158
11	STS (1, 3, 10, 30 mg/kg) 15 minutes before the left anterior descending coronary artery ligation	Male Sprague-Dawley rats	C-Jun N-terminal kinase (JNK)	Reduction of the infarct size, the lactate dehydrogenase level, and the number of apoptotic cardiomyocytes in the infarcted heart	159
12	Imidazole tanshinone derivatives 0.3 μM for 24 hrs	Zebrafish embryos 21 hrs post fertilization	VEGF/FGF-Src-MAPK and PI3K-P38	Increased angiogenesis, vascular protective and restorative activity	160
13	Intragastrically administered STS 10 or 20 mg/kg/day	Male ApoE$^{-/-}$ mice	NLRP3 inflammasome, AMPK-dependent mitochondrial pathway	Inhibits cholesterol crystallization –induced Vascular Endothelial Cell Pyroptosis,	161
14	TIIA (10 mg/kg, 50 mg/kg), dissolved in 0.1% solution of DMSO and administered by gavage to pregnant mice	C57BL/6 mice	GSK-3β	TIIA the amniotic fluid volume in pregnant mice, without the effects on embryo numbers per litter, atrophic embryo rate, fetal weight, and placental	162
15	TIIA intraperitoneal injection, 20 mg/kg	Male apolipoprotein E-deficient (ApoE -/-) mice and male C57BL/6 J mice	COX-2/TNF-a/NF-κB	Attenuation of the plaque buildup and the accumulation of lipids in ApoE-/- mice	45
16	TIIA derivative: (2-(((4-propionyl)oxy)methyl)naphtho[1,2-b]furan-4,5-dione) known as TB3 was given intragastrically (50 mg/kg), every other day for 2 weeks	Male BALB/c-null mice	Androgen receptor-dependent reduction of pyruvate kinase M2, enzyme expression, inhibits the glycolysis cycle and retards the cell growth rate	Inhibition of castration resistant prostate cancer cell lines (CRPC) growth rate *in vitro* and *in vivo* after subcutaneous xenotransplantation to nude mice	163
17	TIIA 5 or 20 mg/kg of administered intraperitoneally once daily for 4 wk	Mice	(NEAT1)/microRNA (miRNA, miR)-291a-3p/member RAS oncogene family Rab22a	Improved neuronal morphology and attenuated oxidative stress and neuroinflammation in the brain tissue of Alzheimer disease mice model	164
